# The impact of frailty on healthcare utilisation in Ireland: evidence from the Irish longitudinal study on ageing

**DOI:** 10.1186/s12877-017-0579-0

**Published:** 2017-09-05

**Authors:** Lorna Roe, Charles Normand, Maev-Ann Wren, John Browne, Aisling M. O’Halloran

**Affiliations:** 1 Centre for Health Policy and Management, Trinity College Dublin, 3-4 Foster Place, College Green, Dublin 2, Ireland; 2 The Economic and Social Research Institute, Whitaker Square, Sir John Rogerson’s Quay, Dublin 2, Ireland; 3 Epidemiology & Public Health, University College Cork, College Road, Cork, Ireland; 4 The Irish Longitudinal Study on Ageing, Trinity College Dublin, College Green, Dublin 2, Ireland

**Keywords:** Frailty, Healthcare utilisation, Complex needs, Ageing, Health and social care planning

## Abstract

**Introduction:**

To examine the impact of frailty on medical and social care utilisation among the Irish community-dwelling older population to inform strategies of integrated care for older people with complex needs.

**Methods:**

Participants aged ≥65 years from the Irish Longitudinal Study on Ageing (TILDA) representative of the Irish community-dwelling older population were analysed (*n* = 3507). The frailty index was used to examine patterns of utilisation across medical and social care services. Multivariate logistic and negative binomial regression models were employed to examine the impact of frailty on service utilisation outcomes after controlling for other factors.

**Results:**

The prevalence of frailty and pre-frailty was 24% (95% CI: 23, 26%) and 45% (95% CI: 43, 47%) respectively. Frailty was a significant predictor of utilisation of most social care and medical care services after controlling for the main correlates of frailty and observed individual effects.

**Conclusions:**

Frailty predicts utilisation of many different types of healthcare services rendering it a useful risk stratification tool for targeting strategies of integrated care. The pattern of care is predominantly medical as few of the frail older population use social care prompting questions about sub-groups of the frail older population with unmet care needs.

**Electronic supplementary material:**

The online version of this article (doi:10.1186/s12877-017-0579-0) contains supplementary material, which is available to authorized users.

## Background

Advances in health technologies and improvements in standards of living have resulted in significant longevity gains. In 1916, over half of the Irish population died before reaching 65 years of age, most commonly from infectious diseases. A century later, eight in ten deaths occur in old age and chronic non-communicable diseases are the leading cause of death [1]. Today’s management of acute health episodes, previously deemed life-threatening, results in many older people surviving into their eighties and nineties [2, 3]. However, epidemiological and demographic changes like this challenge healthcare systems, specifically; managing older people presenting with complex combinations of chronic conditions, geriatric conditions and disabilities [4, 5]. Healthcare systems have been designed traditionally to manage everyday health problems in general practice and acute health events in hospital, resulting in complex entitlement structures and fragmented care delivery for those patients who require care from both sectors. Additionally, patients report difficulties navigating, activating and managing services needed to meet their medical and personal-care needs often over a number of years [6–10]. ‘Integrated care’ has been proposed as a solution to manage this type of problem, viewed as a strategy to improve the patient experience through better coordination of services across all service boundaries [11]. It is a prominent health policy goal [12–14], however, it is recognised as a resource-intensive effort and thus commonly targeted to people with complex needs who require different types of services [15, 16].

Targeting older people with complex needs is a priority as older people are recognised as intensive users of healthcare services. However, conceptualising those older people with complex ‘breadth and depth’ to their needs is challenging. Chronological age alone is not a reliable predictor of healthcare utilisation. Likewise, disability has been found to increase the odds of using social care but not medical care [17] while multimorbidity has been found to increase the odds of using medical care but not social care [18]. The concept of frailty may bridge this gap and act as a lens for complex needs in old age.

Frailty is recognised as a multidimensional geriatric condition, characterised by a decreased reserve and associated with increased risk of adverse outcomes such as falls, hospitalisation, nursing home admission and death, when encountering minor stressors [19]. Frailty is related to, but distinct from disability and multimorbidity [20]. It is a common condition, with prevalence in the community-dwelling population aged ≥65 years ranging from 4% to 59% [21]. It is associated with increasing age which has implications for country’s with an ageing demographic, such as Ireland [22]. In practice, it is recommended that frailty is identified and managed with care planning, the activation and ongoing management of an integrated package of services [23–27]. Many early pilot studies of integrated care targeted older people with frailty [28, 29]. However, it is not clear how to scale these local pilots to national level, as these pilots were designed and implemented without using evidence on the frail older population nationally, their current patterns in service utilisation and whether it was frailty that determined high levels of service utilisation or other factors.

As countries focus their strategy for integrated care on those patients with “complex needs” it is important that such strategies are progressed in a systematic manner with a strong evidence base. Currently little is known about the frail older population in Ireland and their current patterns of service utilisation. Results of studies among community-dwelling older people in other countries indicate frailty has an important role in explaining variation in service use [30–38]. However, the evidence is limited by the multitude of frailty measures in use, reflecting the lack of an agreed standard definition and conceptualisation of frailty in the literature [27]. Two approaches, a frailty-phenotype and a cumulative deficits approach are commonly used [39]. While phenotype frailty is more frequently used in population-based studies [40, 41], both approaches are similar with respect to their strong predictive value for negative outcomes [39] but, different in that each classifies different individuals as frail with only partial overlap [42, 43]. The aim of this study is to examine the relationship between frailty and service utilisation after controlling for confounders such as other healthcare needs, entitlements and socio-economic factors. In so doing, this study will improve our understanding of the potential burden of frailty on healthcare systems as population’s age.

## Methods

This cross-sectional study was based on data from wave one of The Irish Longitudinal Study on Ageing (TILDA), a prospective cohort study. TILDA collects data from a representative sample of the Irish community-dwelling population aged ≥50 years and covers several aspects of social, economic, life-style, physical health and healthcare utilisation. In TILDA, participants are selected according to a multistage sampling design, including stratification, clustering and systematic sampling, which has been described extensively elsewhere [44]. The resulting sample is self-weighting except for biases caused by non-random variations in response rates. These biases have been dealt with at the analysis stage by means of calibration weights.

Data were collected over a 17-month period from October 2009 to February 2011 through a computer assisted personal interview, a self-completed questionnaire and a nurse-led health assessment. Respondents were required to provide written informed consent to participate in the study which may have resulted in the exclusion of those with severe cognitive impairment. In total, 8175 individuals aged ≥50 years were interviewed at baseline. We restricted our analysis to the older adult population aged ≥65 years (*n* = 3507) as frailty is more prevalent in this age-group and 65 years is the minimum age at which one can apply for *Services for Older People* in the Irish healthcare system.

Respondents reported their utilisation of a range of healthcare services in the 12 months preceding the survey, see Additional file 1. Dichotomous variables captured if a respondent used a range of community-based services; dietician, respite, chiropody, physiotherapy, hearing, social work, psychological, homecare (the home help service and personal care service), day centre, optician, dental, community nurse/public health nurse (PHN), occupational therapy (OT), meals on wheels, speech and language therapy. Count variables captured the frequency with which respondents visited the general practitioner (GP), emergency department (ED), outpatient clinic and the number of day case procedures, hospital overnight admissions and nights spent in a hospital.

Phase 3 of the *Anderson and Newman Behavioural Model of Health Service Use* [45] guided the selection of independent variables to be included as potential confounders. The following variables, selected by availability and theoretical reasoning were grouped as predisposing, enabling and need factors.

### Need factors

Independent variables reflecting a need for care included frailty, disability, multimorbidity, falls, self-reported physical health and self-reported emotional health.

Three frailty measures were constructed for use in the first step in the analysis. A cumulative deficits approach was operationalised with the construct of a frailty index (FI) [46] adapted to the TILDA database [47, 48]. The deficits included any symptom, sign, disease, disability or laboratory abnormality associated with age and adverse outcomes, present in at least 1% of the population, covering several organ systems and which had under 5% missing data [49]. A 32-item index was constructed using self-report health measures and categorised into robust (FI score: <0.09374), pre-frail (FI score: 0.09375–0.2499) and frail (FI score: ≥0.25) based on index scores. Respondents missing no more than 20% of deficits were included in the analysis resulting in 3507 participants with a frailty index classification score. Additionally, two phenotype approach measures were operationalised in TILDA [50, 51]. Five objective measures; gait speed, exhaustion, physical inactivity, unintentional weight loss and grip strength were used to construct a Fried Phenotype measure [20]. Fewer respondents underwent a nurse-led health assessment, resulting in 2287 participants with a Fried Phenotype classification score. Finally, the FRAIL scale [52] was operationalised using self-reported responses on five items; fatigue, resistance, ambulation, illness and loss of weight resulting in 2827 participants with a FRAIL scale classification score. Full details of the frailty measures are provided in Additional file 2.

Disability status was measured by combining responses to the Activities of Daily Living (ADL) [53] and the Instrumental Activities of Daily Living (IADL) scales in which individuals with at least one ADL or IADL difficulty were classified as ‘disabled’. Self-reported health status was assessed by asking respondents to rate their health relative to others of the same age and to rate their emotional or mental health, with responses dichotomised into “excellent, very good, good” and “fair, poor”. Respondents were asked if they had fallen in the previous 12 months. Multimorbidity was conceived as the co-occurrence of multiple chronic or acute conditions within one person [54] and operationalized as threshold-multimorbidity indicated by two or more of the following conditions; self-reported poor vision; self-reported poor hearing; high blood pressure or hypertension; angina; heart attack; congestive heart failure; diabetes or high blood sugar; stroke (cerebral vascular disease); mini-stroke/ transient ischemic attack; high cholesterol; heart murmur; heart arrhythmia; chronic lung disease; asthma; arthritis (including osteoarthritis, or rheumatism); osteoporosis; cancer; Parkinson’s disease; any emotional, nervous or psychiatric problem such as depression or anxiety; alcohol or substance abuse; Alzheimer’s disease; dementia, organic brain syndrome, senility; serious memory impairment; stomach ulcers; varicose ulcers; cirrhosis, or serious liver damage. A variable was generated indicating the presence of multimorbidity based on these conditions.

### Predisposing factors

Predisposing factors included age, gender, marital status and living arrangement. Living arrangement was transformed into a dichotomised variable for “living alone” and “living with spouse or others”.

### Enabling factors

Enabling characteristics are described as the means individuals have at their disposal to avail of services and included; education, healthcare entitlement, private health insurance, informal care, transport and household location.

Respondents were asked to indicate the highest level of education that they had completed which was classified as “primary”, “secondary” and “third level or higher”. Healthcare entitlement was assessed by asking respondents about their entitlement to a medical card/GP-visit card and if they had purchased private health insurance. A medical card provides free GP care, eligibility for publicly provided community services and subsidised prescribed medicines while the GP visit card provides free GP care only. Private health insurance in Ireland can be typically used to purchase quicker access to hospital care. A categorical variable was generated indicating those older people with “no cover”, “private health insurance only”, “medical/GP card only” and “dual cover”. A binary variable for the availability of informal help was generated by asking family respondents if, in the last 2 years, they or their spouse/partner had received any practical household help and help with paperwork from non-resident children or grandchildren, other relatives or neighbours and friends. The availability of transport was identified by asking respondents about the type of transport used regularly in the previous 12 months. To drive or to be driven was the most common answer and the variable was transformed to reflect those who could drive or be driven.

Processes of statistical analysis included developing estimates of the dependent healthcare variables which were cross-tabulated with frailty to investigate basic patterns in the data and detect cells with low numbers. These patterns were tested using Pearson’s chi square statistic for the dichotomized dependent outcomes and Krustal-Wallis test for the non-normally distributed count dependent outcomes. Tests were two-tailed, with α threshold of 0.05 for statistical significance. Basic patterns between frailty and health and social care outcomes were examined across three quasi-continuous measures of frailty; the frailty index, Fried phenotype and the FRAIL scale. All three frailty measures displayed statistically increased rates of service utilisation among the frail compared to the pre-frail and robust groups. The conceptualisation and operationalisation of frailty using the cumulative deficits approach, the Frailty Index, was associated with proportionately higher rates of service utilisation in contrast to the Fried and FRAIL scale, see Additional file 3. As a result, the frailty index was chosen for use in this study.

Next, regression techniques were used to test the relationship between frailty and service use outcomes after controlling for other factors. Logistic regression was used to model the impact of frailty on the dichotomized service outcomes and the results were presented as odds ratios. Seven of the fifteen dichotomous dependent variables had sufficient power for the analysis based on a guide of 10 cases required per independent variable to avoid a type II error. Consequently eight variables capturing utilisation of speech and language therapy, hearing, social work, psychological, meals on wheels, day centre, dietician and respite services which were reported in the summary analysis were dropped from the regression modelling. In modeling the utilisation of count variables, ordinary least squares (OLS) regression is not advisable because count data often violate the assumption of normality. It is common in health services research to encounter semi-continuous data which are characterized by a point mass at zero followed by a right-skewed continuous distribution [55]. Consequently, count data econometric methodologies, which assume a skewed, discrete distribution and restrict predicted values to non-negative values, are usually employed in modeling healthcare utilisation [56]. Two non-parametric methodologies; a negative binomial and poisson regression were considered. The dispersion of the data is one criterion for deciding between a poisson and a negative binomial model as a poisson distribution assumes that the conditional mean is equal to the conditional variance [57]. Information criteria and log-likelihood values were used to choose the most appropriate model specification [56, 57] where in each of the six models; lower akaike information criterion (AIC) values indicated that the negative binomial model was the more appropriate model for use and the results were presented as marginal effects.

A multivariate regression modeling strategy was developed based on the underlying conceptual framework. A correlation analysis was run between all independent variables; the results of which identified problematic collinearity between informal care and disability (correlation score: 0.75) and marital status and living arrangements (correlation score: 0.82). Informal care and marital status were thus dropped from the modeling. Each independent variable was examined as significant confounders in each bivariate model, tests were two-tailed with α adopted at the less conservative 0.10 significance level. Multivariate models examined included age, sex and those other independent variables which were significant in the bivariate analysis. The Breusch-Pagan test for heteroskedasticity indicated heterogeneity which was corrected by estimating robust standard errors in the negative binomial models. Multicollinearity diagnostics were performed using variance inflation factor analysis and final models were selected based on model fit determined by lower AIC values. Analyses were performed using STATA version 13.

## Results

A total of 3507 participants (mean age: 74.03; 55% women) were included in this analysis. The weighted prevalence of frailty in the TILDA study sample aged ≥65 years using the categorical frailty index, Fried Phenotype and FRAIL scale, was 24%, 8% and 5% respectively. Disability was experienced by 19.5% of those aged ≥65 years and the prevalence of multimorbidity was 66.7% among this population, see Table 1. Just over one third of respondents lived alone. More than half of the respondents had primary school education only. Most respondents (79.3%) had access to free GP care through the medical card/GP visit card scheme. The majority (88.22%) had access to personal transport by driving or being driven. 12.6% of the older population reported having an informal carer for help with practical household tasks and paperwork.

**Table 1:** Characteristics of the TILDA sample aged ≥65 years (TILDA, wave 1)

	General population ≥ aged 65 years	
Variable	Sub-category	% (95% CI)
Frailty (FRAIL scale)	Robust	61.71 (59.77, 63.62)
	Pre-frail	31.96 (30.13, 33.85)
	Frail	6.33 (5.33, 7.50)
Frailty (Fried phenotype)	Robust	48.48 (46.23, 50.74)
	Pre-frail	43.41 (41.15, 45.69)
	Frail	8.11 (6.84, 9.58)
Frailty (Frailty Index)	Robust	29.89 (28.14, 31.69)
	Pre-frail	45.43 (43.67, 47.12)
	Frail	24.68 (22.99, 26.45)
Multimorbidity	Present	68.1 (66.23, 69.92)
Disability	Present	19.55 (18.05, 21.13)
Self-reported physical health in comparison to others of a similar age	Fair/Poor	17.99 (16.49, 19.6)
Self-reported emotional health	Fair/Poor	10.15 (9.01, 11.41)
Falls	Present	21.66 (20.13, 23.26)
Age	Average years	74.03 (73.74, 74.32)
Sex	Female	55.06 (53.62, 56.50)
Living arrangement	Alone	34.26 (32.49, 36.07)
Healthcare entitlement	No cover	3.73 (3.09, 4.48)
	Private health insurance only	16.94 (15.50, 18.49)
	Medical card only	49.74 (47.31,52.18)
	Dual cover	29.59 (27.59, 31.68)
Education	Primary	56.58 (54.44, 58.70)
	Secondary	31.11 (29.37, 32.91)
	Third level or higher	12.3 (11.20, 13.50)
Household location	Urban	50.88 (46.78, 54.96)
Transport	Available to drive/driven	88.22 (86.58, 89.69)
Informal care	Available informal care	12.61 (11.34, 14.00)
Healthcare utilisation in previous 12 months	Public Health Nurse	12.12 (10.85, 13.51)
	Occupational therapy	2.07 (1.60, 2.67)
	Chiropody	9.04 (7.84, 10.40)
	Physiotherapy	6.29 (5.48, 7.20)
	Speech and language therapy	0.28 (0.15, 0.51)
	Social work	0.26 (0.12, 0.53)
	Psychological	0.47 (0.30, 0.75)
	Meals on wheels	1.95 (1.44, 2.63)
	Daycentre	2.34 (1.85, 3.03)
	Optician	18.06 (16.47, 19.77)
	Dental	11.69 (10.42, 13.10)
	Hearing	3.06 (2.48, 3.78)
	Dietician	1.81 (1.40, 2.33)
	Respite	0.75 (0.47, 1.17)
	Homecare	8.24 (7.17, 9.44)
	General Practitioner visits (dichotomous)	93.84 (92.93, 94.64)
	Emergency Department visits (dichotomous)	16.11 (14.81, 17.5)
	Outpatient clinic visits (dichotomous)	43.92 (41.94, 45.91)
	Day case procedures (dichotomous)	17.72 (16.31, 19.26)
	Hospital admissions (dichotomous)	15.86 (14.63, 17.16)
	Night in hospital (dichotomous)	15.57 (14.36, 16.88)

93.8% of community-dwelling older people aged ≥65 years visited their GP at least once in the previous year. Rates of utilisation were lower across all other services. The second most frequently used service; hospital outpatient clinics, were utilised by 43.9% of the older population. The least frequently used service was the social work service which was used by 0.26% of the older population. Less than 1 in 10 of the older population used services to support them to remain at home including homecare, respite care, meals on wheels and day centre care, see Table 1.

**Fig. 1:**
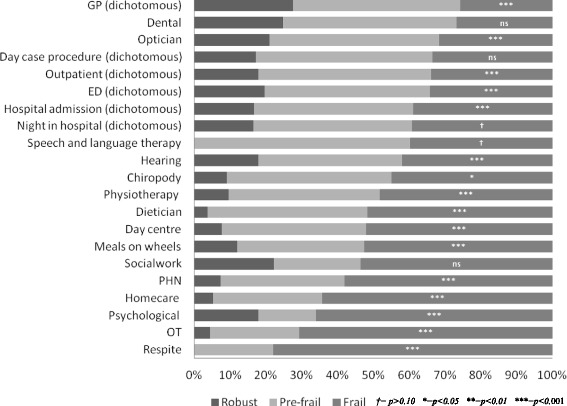
Weighted estimate of frailty among healthcare service users aged ≥65 years (TILDA, wave 1)

Frailty was significantly associated with higher rates of utilisation across different types of healthcare services. The frail were the majority users across those services designed to support older people in their home; such as homecare, respite care, occupational therapy and the Public Health Nurse, see Fig. 1 and Table 2.

**Table 2:** Frailty classification among health services users aged ≥65 years in the community (TILDA wave 1)

	Robust *≥aged 65 years*	Pre-frail *≥aged 65 years*	Frail *≥aged 65 years*	Total	
	% *(95% CI)*	% *(95% CI)*	% *(95% CI)*	%	*p-value*
Public Health Nurse	7.46 (5.12, 10.76)	34.54 (29.47, 39.98)	57.99 (52.26, 63.51)	100	<0.001
Occupational therapy	4.48 (1.60, 11.86)	24.82 (15.15, 37.91)	70.7 (57.97, 80.84)	100	<0.001
Chiropody	9.16 (6.32, 13.11)	45.91 (39.68, 52.26)	44.93 (38.79, 51.22)	100	<0.001
Physiotherapy	9.70 (6.50, 14.23)	42.07 (35.45, 48.98)	48.23 (41.28, 55.25)	100	<0.001
Speech and language therapy	0	60.34 (30.96, 83.77)	39.66 (16.23, 69.04)	100	= 0.092
Social work	22.3 (5.37, 59.19)	24.24 (6.76, 58.52)	53.45 (21.99, 82.39)	100	= 0.140
Psychological	17.9 (6.40, 40.98)	16.17 (5.62, 38.45)	65.93 (42.92, 83.28)	100	<0.001
Meals on wheels	12.15 (5.76, 23.81)	35.43 (24.41, 48.25)	52.43 (38.8, 65.70)	100	<0.001
Daycentre	7.78 (3.19, 17.77)	40.31 (29.07, 52.67)	51.90 (39.14, 64.42)	100	<0.001
Optician	21.05 (17.80, 24.72)	47.42 (43.51, 51.36)	31.53 (27.69, 35.63)	100	<0.001
Dental	24.88 (20.93, 29.30)	48.33 (43.44, 53.25)	26.79 (22.54, 31.52)	100	=.0738
Hearing	17.88 (11.82, 26.12)	40.18 (30.56, 50.62)	41.94 (31.89, 52.71)	100	<0.001
Dietician	3.72 (1.16, 11.22)	44.62 (32.19, 57.77)	51.66 (38.61, 64.48)	100	<0.001
Respite	0	22.15 (9.05, 44.87)	77.85 (55.13, 90.95)	100	<0.001
Homecare	5.24 (3.01, 8.96)	30.46 (24.57, 37.06)	64.3 (57.68, 70.41)	100	<0.001
General Practitioner (dichotomous)	27.57 (25.78, 29.43)	46.72 (44.88, 48.57)	25.71 (23.93, 27.57)	100	<0.001
Emergency Department (dichotomous)	19.71 (16.62, 23.22)	46.19 (41.86, 50.57)	34.1 (29.85, 38.62)	100	<0.001
Outpatient clinic visits (dichotomous)	17.88 (15.85, 20.10)	48.29 (45.62, 50.97)	33.83 (31.24, 36.52)	100	<0.001
Day case procedures (dichotomous)	17.25 (14.29, 20.67)	49.28 (45.23, 53.35)	33.47 (29.56, 37.62)	100	= 0.798
Hospital admissions (dichotomous)	16.65 (13.74, 20.03)	44.46 (40.10, 48.91)	38.89 (34.61, 43.35)	100	<0.001
Night in hospital (dichotomous)	16.51 (13.53, 19.99)	44.28 (39.88, 48.78)	39.21 (34.85, 43.75)	100	<0.001

**Fig. 2:**
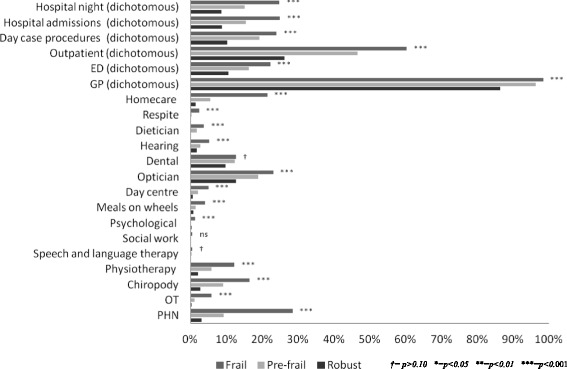
Weighted estimate of healthcare utilisation among robust, pre-frail and frail respondent’s aged ≥65 years (TILDA, wave 1)

**Fig. 3:**
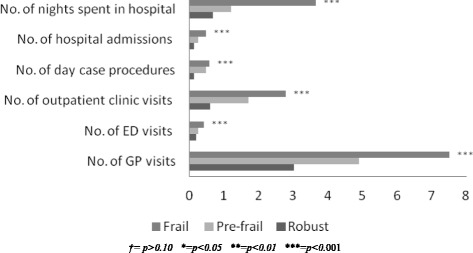
Weighted estimate of frailty and frequency of healthcare utilisation among respondent’s aged ≥65 years (TILDA, wave 1)

While the frail were the dominant users of these services, the proportion of the frail population utilising these services was less than one-third, see Fig. 2 and Table 3, while GP and outpatient clinic care was used by 98% and 60% respectively, of frail older population. Finally, frailty had a statistically significant impact on the average amount of services utilised. While the majority of older people visited their GP in the previous year, frail older people had more visits on average, see Fig. 3 and Table 4. This was also the case for unplanned hospital care, particularly the number of nights spent in hospital. Finally, frailty was associated with higher amounts of outpatient care and day case procedures on average, by comparison to the pre-frail and robust.

**Table 3:** Health service use (previous 12 months) in the community population aged ≥65 years by frailty classification (TILDA wave 1)

	Robust *≥aged 65 years*	Pre-frail *≥aged 65 years*	Frail *≥aged 65 years*	
	% *(95% CI)*	% *(95% CI)*	% *(95% CI)*	*p*-value
Public Health Nurse	3.02 (2.03, 4.49)	9.21 (7.71, 10.96)	28.47 (25.03, 32.18)	<0.001
Occupational therapy	0.31 (0.11, 0.88)	1.13 (0.66, 1.91)	5.94 (4.41, 7.96)	<0.001
Chiropody	2.77 (1.88, 4.07)	9.14 (7.57, 10.98)	16.46 (13.58, 19.80)	<0.001
Physiotherapy	2.04 (1.34, 3.08)	5.83 (4.70, 7.20)	12.3 (10.22, 14.73)	<0.001
Speech and language therapy	0	0.38 (0.17, 0.81)	0.46 (0.17, 1.16)	= 0.092
Social work	0.19 (0.04, 0.79)	0.14 (0.04, 0.46)	0.56 (0.19, 1.61)	= 0.140
Psychological	0.28 (0.10, 0.80)	0.17 (0.05, 0.48)	1.27 (0.71, 2.26)	<0.001
Meals on wheels	0.79 (0.37, 1.68)	1.52 (0.98, 2.35)	4.14 (2.72, 6.25)	<0.001
Daycentre	0.62 (0.24, 1.53)	2.11 (1.44, 3.06)	5.00 (3.52, 7.04)	<0.001
Optician	12.72 (10.54, 15.28)	18.85 (16.73, 21.17)	23.07 (20.06, 26.39)	<0.001
Dental	9.73 (7.81, 12.07)	12.44 (10.81, 14.28)	12.69 (10.47, 15.31)	= 0.073
Hearing	1.83 (1.17, 2.84)	2.71 (1.96, 3.72)	5.21 (3.72, 7.24)	<0.001
Dietician	0.22 (0.07, 0.71)	1.78 (1.22, 2.56)	3.79 (2.58, 5.53)	<0.001
Respite	0	0.36 (0.14, 0.89)	2.36 (1.41, 3.94)	<0.001
Homecare	1.45 (0.81, 2.55)	5.52 (4.33, 7.02)	21.47 (18.38, 24.91)	<0.001
General Practitioner visits (dichotomous)	86.35 (84.09, 88.33)	96.3 (95.14, 97.19)	98.42 (97.03, 99.16)	<0.001
Emergency Department visits (dichotomous)	10.62 (8.89, 12.63)	16.37 (14.49, 18.45)	22.3 (19.31, 25.61)	<0.001
Outpatient clinic visits (dichotomous)	26.22 (23.37, 29.27)	46.68 (44.09, 49.28)	60.36 (56.3, 64.27)	<0.001
Day case procedures (dichotomous)	10.23 (8.34, 12.47)	19.25 (17.32, 21.3)	24.02 (20.89, 27.46)	<0.001
Hospital admissions (dichotomous)	8.83 (7.26, 10.71)	15.51 (13.72, 17.48)	25 (22.01, 28.26)	<0.001
Night in hospital (dichotomous)	8.59 (7.04, 10.45)	15.18 (13.39, 17.13)	24.78 (21.79, 27.98)	<0.001

**Table 4:** Average use of health services in the previous 12 months among adults aged ≥65 years by frailty classification (TILDA wave 1)

	*Population ≥ aged 65 years*	Robust *≥aged 65 years*	Pre-frail *≥aged 65 years*	Frail *≥aged 65 years*	
Number of	*Average no. (95% CI)*	*Average no. (95% CI)*	*Average no. (95% CI)*	*Average no. (95% CI)*	*p*-value
General Practitioner visits	4.99 (4.76, 5.21)	3.03 (2.82, 3.24)	4.91 (4.67, 5.15)	7.51 (6.89, 8.14)	<0.001
Emergency Department visits	0.27 (0.23, 0.30)	0.19 (0.12, 0.25)	0.24 (0.19, 0.28)	0.42 (0.33, 0.50)	<0.001
Outpatient visits	1.64 (1.49, 1.79)	0.6 (0.49, 0.72)	1.71(1.49, 1.93)	2.80 (2.34, 3.20)	<0.001
Day case procedures	0.40 (0.33, 0.47)	0.13 (0.10, 0.16)	0.48 (0.35, 0.60)	0.58 (0.41, 0.76)	<0.001
Hospital admissions	0.27 (0.23, 0.30)	0.12 (0.09, 0.15)	0.25 (0.19,0.30)	0.48 (0.39, 0.58)	<0.001
Nights spent in hospital	1.64 (1.39, 1.89)	0.67 (0.45, 0.88)	1.20 (0.95, 1.45)	3.65 (2.83, 4.46)	<0.001

Results for the impact of frailty on each of the seven community based dichotomized outcomes are presented in Table 5. In the unadjusted models, frailty was found to be a significant predictor of utilising these services. The impact of frailty is particularly notable with respect to the odds of utilising the homecare service (OR: 20.96) and the PHN (OR: 14.60). The effect of frailty is reduced in the adjusted models as other factors known to be determinants of service use are added to the model. Despite the inclusion of factors such as disability and healthcare entitlement, a strong effect of frailty on the homecare service (OR: 7.4) was observed. In the multivariate analysis, no statistically significant impact of frailty on the adjusted odds of using optician (*p* = 0.054), dental (*p* = 0.199) and hearing (*p* = 0.099) services was detected.

**Table 5:** Bivariate and multivariate logistic regression model for community-based service utilisation among adults aged ≥65 years (TILDA wave 1)

	Unadjusted Odds Ratio			Adjusted Odds Ratio		
	n	Pre-frail	Frail	n	Pre-frail	Frail
Public Health Nurse^a^	3507	3.61***	14.60***	3485	2.07**	3.95***
Chiropody^b^	3507	3.42***	7.46***	3479	2.20**	2.86***
Physiotherapy^c^	3507	2.74***	6.61***	3491	2.31**	3.90***
Homecare^d^	3507	4.10***	20.96***	3486	2.75**	7.39***
Optician^e^	3507	1.61***	2.11***	3487	1.28	1.41
Dental^f^	3507	1.37*	1.46**	3486	1.18	1.27
Hearing^g^	3507	1.50	2.87***	3364	1.18	1.70

* = *p* < 0.05, ** = *p* < 0.01, *** = *p* < 0.001

*The independent variables entered into each service outcome model were:*

^a^Frailty, disability, multimorbidity, physical health, falls, age, gender, living arrangements, healthcare entitlement, transport, education

^b^Frailty, disability, multimorbidity, physical health, emotional health, falls, age, gender, living arrangements, healthcare entitlement, transport, education, household location

^c^Frailty, disability, multimorbidity, physical health, falls, age, gender, living arrangements

^d^Frailty, disability, multimorbidity, physical health, emotional health, age, gender, living arrangements, healthcare entitlement, education

^e^Frailty, disability, multimorbidity, physical health, age, gender, living arrangements, healthcare entitlement, education

^f^Frailty, multimorbidity, falls, age, gender, healthcare entitlement, education, household location

^g^Frailty, disability, falls, age, gender, healthcare entitlement

Results for the impact of frailty in each of the six medical service count outcomes in the final model are presented in Table 6.The unadjusted marginal effects for using GP and hospital-based services were significantly higher for frail compared with robust participants (reference category). This was particularly so with respect to the marginal effects of frailty on frequency of GP visits where frailty was estimated as resulting in a marginal increase of 4.4 visits to the service. Frailty also had a large impact on the number of nights a respondent spent in hospital where the marginal effect of frailty was estimated as an additional 3.11 nights in comparison to the robust group. The effect of frailty found in the bivariate modelling was reduced after controlling for other need, enabling and predisposing variables. Adjusted marginal effects indicate that the frail participants had 2.29 times more GP visits, 1.50 times more outpatient visits, 0.23 times more hospital admissions, 0.41 times more day case procedures and 1.33 times more nights in a hospital in the previous 12 months in comparison to the robust category. Visits to the ED (*p* = 0.27) was no longer statistically significant in the multivariate analysis.

**Table 6:** Bivariate and multivariate negative binomial regression models for GP and hospital service utilisation among those adults aged ≥65 years (TILDA wave 1)

	Unadjusted marginal effect			Adjusted marginal effect		
	n	Pre-frail	Frail	n	Pre-frail	Frail
General Practitioner visits^a^	3501	1.84***	4.40***	3471	1.35***	2.29***
Outpatient clinic visits^b^	3504	1.17***	2.32***	3476	0.94***	1.50***
Emergency Department visits^c^	3504	0.05	0.24***	3487	−0.001	0.06
Hospital admissions^d^	3505	0.12***	0.41***	3481	0.10**	0.23***
Day case procedures^e^	3503	0.37***	0.54***	3485	0.30***	0.41***
Nights spent in hospital^f^	3504	0.54**	3.11***	3485	0.30*	1.33***

* = *p* < 0.05, ** = *p* < 0.01, *** = *p* < 0.001

*The independent variables entered into each service outcome model were:*

^a^Frailty, disability, multimorbidity, physical health, emotional health, falls, age, gender, living arrangements, healthcare entitlement, education, household location

^b^Frailty, disability, multimorbidity, physical health, emotional health, falls, age, gender, healthcare entitlement, household location

^c^Frailty, disability, multimorbidity, physical health, emotional health, falls, age, gender

^d^Frailty, disability, multimorbidity, physical health, falls, age, gender, healthcare entitlement, household location

^e^Frailty, disability, multimorbidity, physical health, emotional health, age, gender, healthcare entitlement

^f^Frailty, disability, multimorbidity, physical health, emotional health, falls, age, gender, healthcare entitlement

## Discussion

We found that frailty, measured with the frailty index, has an important role to play in explaining variation in service utilisation in Ireland, even when controlling for other health and socio-economic indicators. This is broadly consistent with the results of other research [30, 33, 35]. Consequently, there is strong evidence to recommend the addition of frailty as an important ‘need’ variable in the Behavioural Model of Health Service Use in future studies as it captures previously unexplained variation in service utilisation across a range of different types of services.

The findings from this study indicate that general practice is a prominent service in the management of frailty. This study found that frailty results in a significant increase in the number of times an older person visited their GP in the previous year. This result was similarly found in a Europe-wide study [31]; in a Belgian study [30] and finally in an Australian study of men aged ≥70 years [32]. As each of these studies used different measures of frailty to this study, and different combinations of confounding variables, within slightly different populations, direct comparison of the magnitude of the effect is difficult.

However, the largest effect of frailty is experienced in the homecare service where an individual who is frail is nearly twice as likely to receive a homecare service than someone who is pre-frail, and over eight times more likely to receive a homecare service than a robust individual. This effect of frailty on homecare has been found in another study in the Belgian context [30]. However frailty status alone did not solely determine the receipt of homecare services, and factors such as living alone and increasing age are significant determinants of homecare utilisation which is in keeping with the findings in the Irish context [17, 58]. This study also found that frailty is a significant predictor of physiotherapy utilisation which is in keeping with the findings of a study of men aged ≥70 living in Sydney [32].

Frailty had a significant impact on variation in patterns of unplanned hospital care; specifically hospital admissions and nights spent in hospital. These findings are in keeping with those observed in the Belgian study [30] and in a US study which found that a higher number of frailty deficits was associated with greater risk of hospitalization [33]. However, a significant relationship between frailty and the ED utilisation after adjusting for known confounders was not detected. This contrasts with findings in the Belgian study which found that the differences between frail and robust older people were most pronounced in their contacts with the ED [30]. Although this was not found in the Irish context among a similarly aged population, most of the variation in the analysis presented here was captured by disability, self-reported health and falls; variables which were not included in the Belgian model but which may reflect the unplanned acute health events which commonly lead to ED visits. However, frailty was found to be a significant predictor of length of stay in a hospital, in keeping with findings from an Australian study [32].

Finally, this study found a statistically significant association between frailty and planned hospital care; specifically visits to an outpatient clinic and any medical procedures which were carried out on as a day-case procedure. This contrasts with a US study which did not find a statistically significant relationship between frailty and outpatient ED visits. The researchers concluded that their findings suggested that repeat outpatient ED visits are a unique type of health service utilisation that non-health-related factors may influence more significantly than a global measure of health [33]. However, their study was not representative of the population aged ≥65 in the United States but of a *subsample* of this age-group who had previously visited the ED and there are also differences between the classification of outpatient services in the studies making a comparison unsuitable.

Overall, the effect of frailty found in this study is interesting in the Irish context as a frailty classification is not currently used as a criterion in the service allocation process for older people. This validates the viewpoint that frailty can be subjectively identified in clinical practice [59] as reflected in service allocation patterns identified here. However, most of the frail older population fail to utilise social care services, which indicates a potential weakness in the targeting of these services in Irish long term care system, which has been discussed elsewhere [17]. These findings also raise questions about potential sub-groups of the frail older population who are failing to utilise these services which can be investigated within the TILDA database using person-centred analytical techniques.

### Study strengths and weaknesses

This study is limited by the cross-sectional design which allows the interpretation of associations rather than causation. Secondly, the impact of frailty on healthcare utilisation may be underestimated as this study does not include older people in convalescent care or inpatient hospital care who were excluded during the first wave of data collection. This limitation will be overcome in subsequent waves. Thirdly, this study used both the self-report dependent and self-report independent variables which raises an issue with common method variance [60], however this is counteracted with face-to-face computer aided interview technique and strong survey design. Fourthly, the potential for measurement error in counting service utilisation events is well-recognised [61]. Specifically it is known that under-reporting of utilisation is exacerbated by increased utilisation [62]. While we acknowledge this potential limitation of our dependent variables, it must be remembered that surveys of this kind are the usual source of data for analyses of this type. Finally, factors associated with service utilisation such as the availability of services or distances to services were beyond the scope of this study and could therefore be considered a limitation. The strengths of this study include the large sample size which is generalisable to the Irish population, a strong questionnaire design and data collection processes and the contribution of knowledge about frailty and service utilisation in Ireland.

## Conclusion

This study has identified a sub-group of the older population, classified as frail according to their score on the frailty index, who were identified as heavy-users of healthcare services. This frail sub-group use more of medical and social care services in comparison to those who are pre-frail or robust even after controlling for many factors known to drive service use. Frailty is clearly an important and useful concept of need capturing those older individuals who are users of many different services. Going forward, it will be important to develop strategies for integrated care targeted to support frail older people so that they can receive the right combinations of services, in the right place at the right time which can support them to remain living in their own home. Key to informing such a strategy will be the identification of sub-groups of the frail older population who are not accessing such supports and experiencing poor outcomes.

## Additional files

Additional file 1: Details of dependent variables from the TILDA CAPI questionnaire. (DOCX 13 kb)

Additional file 2: Details of independent frailty variables operationalised in the TILDA database using CAPI and health assessment data. (DOCX 14 kb)

Additional file 3: Sensitivity analysis to compare how well each of the three frailty measures capture health care use. (DOCM 38 kb)

## Abbreviations

ADL: Activities of Daily Living
ED: Emergency department
FI: Frailty index
GP: General practitioner
IADL: Instrumental Activities of Daily Living
OT: Occupational therapist
PHN: Public health nurse
TILDA: The Irish Longitudinal Study on Ageing
