# Supplementary material for: The impact of frailty on healthcare utilisation in Ireland: evidence from the Irish longitudinal study on ageing
Source: BMC Geriatr. 2017 Sep 5;17:203. doi: 10.1186/s12877-017-0579-0 (PMC5583758; doi:10.1186/s12877-017-0579-0)
Supplement: Supplementary file 1 — Details of dependent variables from the TILDA CAPI questionnaire. (DOCX 13 kb) [file 12877_2017_579_MOESM1_ESM.docx]

## Description: Details of dependent variables from the TILDA CAPI questionnaire

- In the last 12 months, did you receive any of the following State services? Exclude any services for which respondent pays anything other than a token or nominal amount.
- Public Health or Community Nurse
- Occupational therapy
- Chiropody
- Physiotherapy
- Speech & language therapist
- Social work
- Psychological/counselling
- Home help
- Personal care attendant
- Meals-on-Wheels
- Day centre
- Optician
- Dental
- Hearing
- Dietician
- Respite
- In the last 12 months, about how often did you visit your GP?
- In the last 12 months, how many times did you visit a hospital Emergency Department (sometimes called A&E or Accident and Emergency) as a patient?
- In the last 12 months, about how many visits did you make to a hospital as an out-patient? (Include all types of consultations, tests, operations, procedures or treatments)
- In the last 12 months, about how many visits did you make to a hospital as an outpatient?
- In the last 12 months, on how many occasions were you admitted to hospital overnight? Note: These are sometimes called in-patient admissions.
- In total, about how many nights did you spend in hospital in the last 12 months?
