# Supplementary material for: The impact of frailty on healthcare utilisation in Ireland: evidence from the Irish longitudinal study on ageing
Source: BMC Geriatr. 2017 Sep 5;17:203. doi: 10.1186/s12877-017-0579-0 (PMC5583758; doi:10.1186/s12877-017-0579-0)
Supplement: Supplementary file 2 — Details of independent frailty variables operationalised in the TILDA database using CAPI and health assessment data. (DOCX 14 kb) [file 12877_2017_579_MOESM2_ESM.docx]

## Description: Details of independent frailty variables operationalised in the TILDA database using CAPI and health assessment data

**FRIED phenotype:** 5 indicators based on objective and self-reported measures from TILDA health assessment and CAPI data. Variables included: Gait speed (Timed Up and Go), Exhaustion, Physical Activity, Weight Loss, Grip strength.

The categorical cut-points were: 0: Robust, 1-2: Pre-frail and ≥3: Frail

**Frailty Index:** 32 deficits based on self-reported measures from TILDA CAPI questionnaire Variables included:

1. Difficulty walking 100m
2. Difficulty rising from a chair
3. Difficulty climbing stairs
4. Difficulty stooping, kneeling or crouching
5. Difficulty reaching above shoulder height
6. Difficulty pushing/pulling large objects
7. Difficulty lifting/carrying weights ≥10lb
8. Difficulty picking up a coin from a table
9. Feeling lonely
10. Poor self-rated physical health
11. Poor self-rated vision
12. Poor self-rated hearing
13. Poor self-rated memory
14. Difficulty following a conversation
15. Daytime sleepiness
16. Polypharmacy
17. Knee pain
18. Hypertension
19. Angina
20. Heart attack
21. Diabetes
22. Stroke and transient ischemic attack
23. High cholesterol
24. Irregular heart rhythm
25. Other CVD
26. Cataracts
27. Glaucoma and age-related macular degeneration
28. Arthritis
29. Osteoporosis
30. Cancer
31. Varicose ulcer
32. Incontinence

The categorical cut-points were: FI score <0.09374: Robust, FI score 0.09375-0.2499: Pre-frail and FI score ≥0.25: Frail.

**FRAIL scale:** 5 indicators based on self-reported measures from TILDA CAPI questionnaire data. Variables included:

*Fatigue:* How much of the time during the past 4 weeks did you feel tired?

*Resistance:* By yourself and not using aids, do you have any difficulty walking up one flight of stairs without resting?

*Ambulation:* By yourself and not using aids, do you have any difficulty walking several hundred yards?

*Illnesses:* ≥5 of the following conditions: cancer, heart attack, heart failure, stroke, diabetes, high blood pressure, high cholesterol, cataracts, arthtiris, osteoporosis, lung disease, asthma, Parkinson’s Disease, hip fracture and varicose ulcer.

*Loss of weight:* In the past year have you lost 10 pounds (4.5 kg) or more in weight when you were not trying to?

The categorical cut-points were: 0: Robust, 1-2: Pre-frail and ≥3: Frail.
