# Supplementary material for: The impact of frailty on healthcare utilisation in Ireland: evidence from the Irish longitudinal study on ageing
Source: BMC Geriatr. 2017 Sep 5;17:203. doi: 10.1186/s12877-017-0579-0 (PMC5583758; doi:10.1186/s12877-017-0579-0)
Supplement: Supplementary file 3 — Sensitivity analysis to compare how well each of the three frailty measures capture health care use. (DOCM 38 kb) [file 12877_2017_579_MOESM3_ESM.docm]

# Description: Examining three measures of frailty as a predictor of service utilization”

The data outlining the differences in proportions of service use per each frailty measure were not included in the original submission, but are provided below. To compare frailty measures, each measure was transformed into quasi-continuous variables by examining rates of service utilisation across a decile distribution of each frailty measure. The frailty index was best equipped to make this transformation as prior to categorization, it is an index ranging from (0.0, 0.1, 0.2, 0.3, 0.4…1). By contrast, the Fried phenotype and FRAIL scales prior to categorization are both a sum of positive indications across five indicators (0,1,2,3,4,5) and thus transform to a quantile distribution; see Table 1 below.

**Table 1 Quasi continuous frailty measures operationalized in the TILDA dataset among participants aged ≥65 years**

| *Decile* | *10* | *20* | *30* | *40* | *50* | *60* | *70* | *80* | *90* | *100* |
| --- | --- | --- | --- | --- | --- | --- | --- | --- | --- | --- |
| Quasi-continuous Frailty Index score (n=3,507) | 0.03 | 0.06 | 0.08 | 0.11 | 0.12 | 0.16 | 0.20 | 0.25 | 0.33 | 0.70 |
| Quasi-continuous Fried Phenotype score (n=2,287) | 0 | 0 | 0 | 0 | 0 | 1 | 1 | 1 | 2 | 3-5 |
| Quasi-continuous FRAIL scale score (n=3,486) | 0 | 0 | 0 | 0 | 0 | 0 | 1 | 1 | 2 | 3-5 |

Using these new quasi-continuous variables, we examined the association between each frailty measure and service utilisation. We found that the tenth decile in the quasi-continuous frailty index accounted for between 13-54% of social care or allied health care. By contrast, the fourth quartile in the quasi-continuous FRAIL scale captured 6-30% of social care or allied health care and similarly, the fourth quartile in the quasi-continuous Fried phenotype captured between 1-31% of social care or allied health care utilisation, see Figure 1, 2, and 3 below.

**Figure 1 The association between service utilisation and the quasi-continuous frailty index measure in the older population aged ≥65 years (TILDA, wave 1)**

**Figure 2 The association between service utilisation and the quasi-continuous FRAIL scale measure in the older population aged ≥65 years (TILDA, wave 1)**

**Figure 3 The association between service utilisation and the quasi-continuous Fried phenotype measure in the older population aged ≥65 years (TILDA, wave 1)**

We were also able to look at the association medical care utilisation and the quasi-continuous frailty measures. While the results are less stark, those in decile 10 of the quasi-continuous frailty index used between 12-20% of medical care utilisation by comparison to 8-16% for quartile four in the quasi-continuous Fried phenotype and 5-12% for quartile four in the quasi-continuous FRAIL scale.

**2.2 Choosing one frailty measure to use**

Overall, the association between frailty and service utilisation was significant across all three quasi-continuous frailty measures. Increasing frailty resulted in a significant increase in the rate of service utilisation across many different types of services. However, the largest increase in service utilisation is captured by the top 10% in the frailty index. Furthermore, the increase in rates of utilisation is found from decile 8 onwards. The frailty score corresponding to decile 8 (<0.25) indicate the theoretical threshold used to categorise ‘frailty’ in the literature.

- 1. **If we had used any other measure would we have come to the same conclusion?**

To validate the view that each frailty measure is a predictor of service utilisation we ran a sensitivity analyses with receiver operator curve (ROC) analysis. ROC analysis is used in clinical epidemiology to quantify how accurately medical diagnostic tests can discriminate between two states (Hajian-Tilaki, 2013), in this case “service user” and “not a service user”. We used the area under the curve (AUC) as a summary of the combined measure of sensitivity and specificity that describes the inherent validity of the frailty measures (Hajian-Tilaki, 2013), see Table 2 below. In this analysis, the AUC scores show that each frailty measures has predictive ability to discriminate those who utilise services.

**Table 2 AUC scores as a predictor of service use outcomes across three frailty measures among the sample aged ≥65 years in TILDA, wave 1**

|  | **Frailty Index**  **(n=3,507)** | **Fried phenotype (n=2,286)** | **FRAIL scale**  **(n=3,486)** |
| --- | --- | --- | --- |
| **Public Health Nurse** | 0.73 | 0.72 | 0.72 |
| **Occupational therapy** | 0.77 | 0.67 | 0.71 |
| **Chiropody** | 0.67 | 0.62 | 0.62 |
| **Physiotherapy** | 0.67 | 0.60 | 0.64 |
| **Speech and language therapy** | 0.69 | 0.77 | 0.70 |
| **Social work** | 0.61 | 0.50 | 0.66 |
| **Psychological** | 0.67 | 0.65 | 0.70 |
| **Homecare** | 0.76 | 0.77 | 0.43 |
| **Meals on wheels** | 0.68 | 0.74 | 0.68 |
| **Day centre** | 0.70 | 0.68 | 0.66 |
| **Optician** | 0.57 | 0.55 | 0.55 |
| **Dental** | 0.54 | 0.52 | 0.54 |
| **Hearing** | 0.60 | 0.58 | 0.61 |
| **Dietician** | 0.70 | 0.57 | 0.66 |
| **Respite** | 0.82 | 0.71 | 0.75 |
| **General Practitioner (dichotomous)** | 0.71 | 0.61 | 0.59 |
| **Emergency Department (dichotomous)** | 0.59 | 0.58 | 0.58 |
| **Outpatient clinic (dichotomous)** | 0.65 | 0.54 | 0.58 |
| **Day case procedures (dichotomous)** | 0.60 | 0.53 | 0.56 |
| **Hospital admissions (dichotomous)** | 0.62 | 0.60 | 0.62 |
| **Nights spent in hospital (dichotomous)** | 0.62 | 0.61 | 0.62 |

We also compared the covariance between the three correlated ROC curves to test if there were significant differences in the area under the curve across the three measures, see Table 3.

**Table 3 A comparative analysis of AUC scores for three frailty measures as a predictor of service use outcomes among the sample aged ≥65 years in TILDA, wave 1**

|  | **Frailty Index**  **(n=2,286)** | **Fried phenotype (n=2,286)** | **FRAIL scale**  **(n=2,286)** | **p-value** |
| --- | --- | --- | --- | --- |
| **Public Health Nurse** | 0.73 | 0.72 | 0.71 | =0.6 |
| **Occupational therapy** | 0.77 | 0.68 | 0.67 | <0.01 |
| **Chiropody** | 0.68 | 0.62 | 0.63 | <0.05 |
| **Physiotherapy** | 0.65 | 0.60 | 0.61 | <0.05 |
| **Speech and language therapy** | 0.60 | 0.77 | 0.58 | <0.001 |
| **Social work** | 0.89 | 0.50 | 0.82 | <0.001 |
| **Psychological** | 0.78 | 0.65 | 0.77 | =0.25 |
| **Homecare** | 0.76 | 0.77 | 0.73 | =0.17 |
| **Meals on wheels** | 0.73 | 0.74 | 0.72 | =0.95 |
| **Day centre** | 0.67 | 0.68 | 0.64 | =0.46 |
| **Optician** | 0.57 | 0.55 | 0.54 | <0.05 |
| **Dental** | 0.53 | 0.52 | 0.54 | =0.14 |
| **Hearing** | 0.58 | 0.58 | 0.58 | =0.96 |
| **Dietician** | 0.73 | 0.57 | 0.65 | <0.01 |
| **Respite** | 0.79 | 0.71 | 0.76 | =0.53 |
| **General Practitioner (dichotomous)** | 0.7 | 0.61 | 0.61 | <0.001 |
| **Emergency Department (dichotomous)** | 0.58 | 0.58 | 0.57 | =0.78 |
| **Outpatient clinic (dichotomous)** | 0.63 | 0.54 | 0.57 | <0.001 |
| **Day case procedures (dichotomous)** | 0.59 | 0.53 | 0.55 | <0.001 |
| **Hospital admissions (dichotomous)** | 0.60 | 0.60 | 0.60 | =0.93 |
| **Nights spent in hospital (dichotomous)** | 0.60 | 0.61 | 0.60 | =0.96 |

In this repeated measures analysis, the sample size is reduced to the minimum number of TILDA participants aged 65 years and older, who had a frailty classification across three of the frailty measures (n=2286). Significant differences were detected across many of the services, however, low cell counts resulted in an inability to reject the null hypothesis that the AUC scores were significantly different.

Overall, this analyses validates the point that frailty, measured in different ways, is a predictor of service utilisation. Higher AUC scores were found for the frailty index in many services, confirming the view that the frailty index is a useful measure to use in this study. There are reasons for why we found this. Firstly, the Fried phenotype measure results in a loss of 35% of TILDA participants aged 65 years and older as it is based from objectively measured data from the TILDA Health Assessment. We know from previous analyses on the health assessment and CAPI samples that the health assessment sample is a younger, fitter sample (Cronin et al, 2013; Kearney et al, 2011). It is likely that older people who were very-frail were unable to travel to the health centre to undertake the health assessment and consequently are missing from this sample. Secondly, significant differences were unlikely to have been detected in the trimmed sample (n=2286) due to low cell counts in the “received service”. If we were to perform this analysis on a larger dataset, this problem may be overcome. Overall, we have strong reasons to choose the frailty index for this study, and modest evidence to suggest the frailty index is the best measure to examine service use outcomes.

**References**

Cronin H, O'Regan C, Finucane C, Kearney P, Kenny RA. Health and aging: development of the Irish Longitudinal Study on Ageing health assessment. J Am Geriatr Soc. 2013 May;61 Suppl 2:S269-78

Kearney PM, Cronin H, O'Regan C, Kamiya Y, Whelan BJ, Kenny RA. Comparison of centre and home-based health assessments: early experience from the Irish Longitudinal Study on Ageing (TILDA). Age Ageing. 2011 Jan;40(1):85-90.

Hajian-Tilaki K. Receiver Operating Characteristic (ROC) Curve Analysis for Medical Diagnostic Test Evaluation . Caspian Journal of Internal Medicine. 2013;4(2):627-635.
